# Pneumonia, an Unusual Initial Presentation of Neonatal Herpes Infection

**DOI:** 10.1155/2019/9594289

**Published:** 2019-10-30

**Authors:** Franck Kouadio, Gil Klinger

**Affiliations:** ^1^Neonatal Intensive Care Unit, Schneider Children's Medical Center of Israel, Petach Tikva, Israel; ^2^Tel Aviv University, Sackler School of Medicine, Tel Aviv, Israel

## Abstract

Neonatal herpes simplex virus (HSV) infection is a life-threatening infection with high morbidity and mortality rates. Neonatal herpes, most commonly due to HSV type 2, is a multi-system disease; however, initial pulmonary presentation is extremely unusual. We describe an infant presenting with progressive respiratory distress, which was the dominant clinical feature of HSV infection during the first days of life. Sepsis work-up and antibiotic treatment were immediately initiated; however, antiviral treatment was not given until the infant's death. HSV type 1 was isolated in nasopharyngeal and endotracheal aspirates. HSV pneumonia should be considered in a newborn with respiratory deterioration not compatible with common neonatal respiratory diseases.

## 1. Introduction

Neonatal herpes simplex virus (HSV) infection is considered a major cause of severe perinatal infections [1]. The infection may be transmitted to newborns at different times, intrauterine, perinatal, and postnatal [2]. However, infants are mostly often infected during their passage through the birth canal [3]. Offspring of mothers with primary HSV are at a greater risk of transmission. HSV-1 and HSV-2 can induce eye or skin lesions, meningoencephalitis, disseminated infections, or congenital malformations [4]. These clinical manifestations are classified as follows [5]: (i) disease localized to the skin, eyes, and/or mouth (SEM disease, accounting for ~45% of cases of neonatal HSV); (ii) encephalitis (Central Nervous System (CNS) disease, accounting for ~30% of cases of neonatal HSV); and (iii) disseminated infection involving multiple organs, including the CNS, lungs, liver, adrenal glands, skin, eyes, and/or mouth (disseminated disease, accounting for ~25% of cases of neonatal HSV). The most devastating form is disseminated infection, that has an 85% mortality rate for affected newborns who did not receive early and appropriate treatment [2]. It is widely accepted that the disseminated form generally presents between the 5^th^ and 12^th^ days of life. Signs include irritability, seizures, respiratory distress, jaundice, bleeding diatheses, shock, and frequently vesicular exanthema [1, 2]. It is therefore uncommon to diagnose pulmonary infection due to HSV before the 5^th^ day of life, and also unusual to observe HSV pneumonia as a primary and even isolated entity of the infection.

We report a case of a neonate with unusual early HSV pneumonia without initial systemic manifestations.

## 2. Case Report

A preterm male infant was born at 36 weeks of gestation by normal vaginal delivery with Apgar scores of 9 and 10 at one and five minutes, respectively. On admission, physical examination was normal with a birth weight of 2620 g. A comprehensive review of the mother's pregnancy revealed fetal bilateral hydroureteronephrosis, which was confirmed by renal ultrasound and cystourethrography.

**Figure 1:**
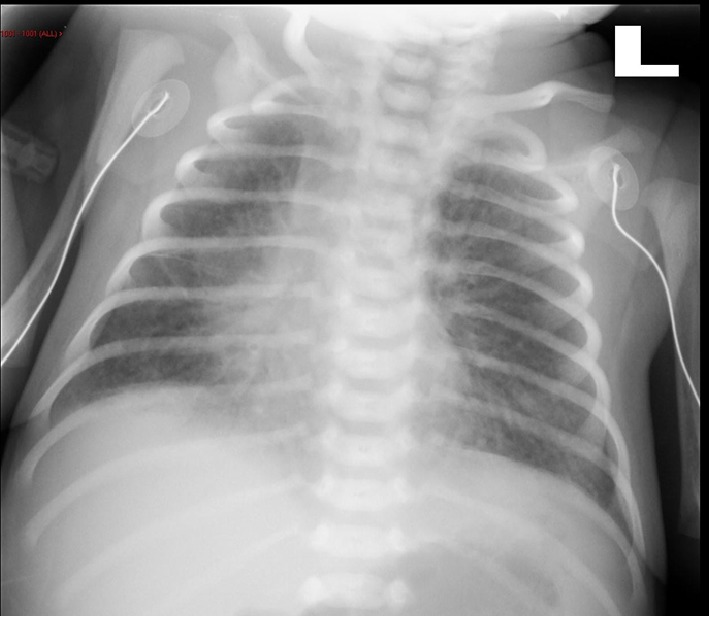
Chest X-ray on day 4. Diffuse interstitial pattern with normal lung volume.

**Figure 2:**
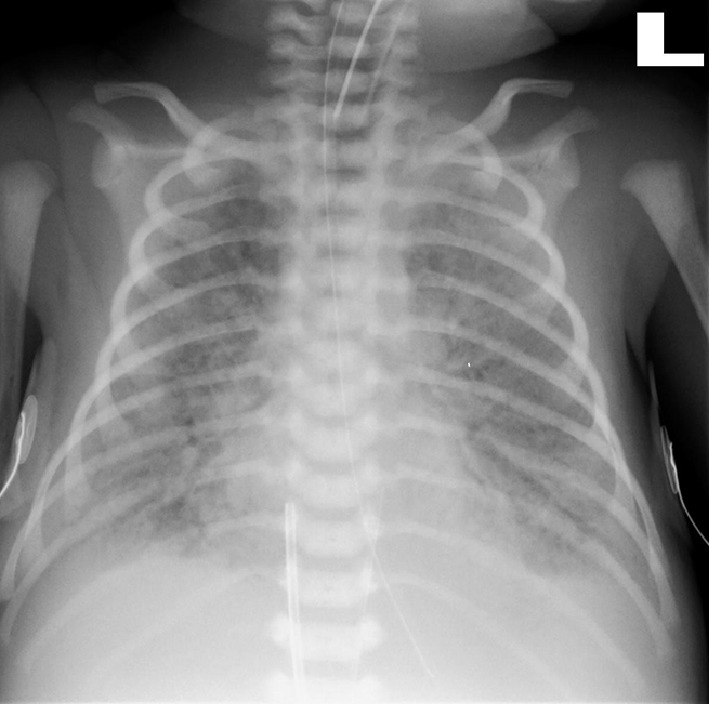
Chest X-ray on day 4. Diffuse alveolar pattern.

On day two, clinical examination was normal and blood samples were obtained in preparation for a retrograde cystography. Mild leukopenia (8500/mm^2^) with lymphopenia (1200/mm^2^) was noticed, with slightly elevated C-reactive protein level (3 mg/dL). The biochemistry revealed a normal creatinine level, and a moderate rise of liver enzymes aspartate aminotransferase (AST) 106 U/L. However, the alanine aminotransferase (ALT) level was normal 20 U/L. Chest X-ray was normal, blood culture was taken, and oral Cephalexin was initiated in preparation for the retrograde cystography. On day 3, an elevated temperature of 38.2°C prompted a full sepsis work-up (including cerebrospinal fluid analysis and culture, complete blood count, biochemistry, blood, and urine cultures). Laboratory tests yielded: a normal CBC with slight lymphopenia 900 cells/mm^3^, a normal biochemistry, CSF with 3 WBCs, a protein level of 77 mg/dL and a glucose level of 51 mg/dL. Intravenous Ampicillin 100 mg/kg/day and Cefotaxime 100 mg/kg/day were initiated. Blood, CSF, and urine cultures were negative. On day 4 of life, signs of respiratory distress including dyspnea and tachypnea of up to 87 breaths/minute were evident. The blood gases revealed hypoxemia (PaO_2_: 35.7 mmHg) and hypercarbia (PaCO_2_: 72.8 mmHg). Respiratory support by nasal continuous positive airway pressure (CPAP) followed by intermittent mechanical ventilation was initiated. A chest X-ray demonstrated bilateral interstitial infiltrates without pleural effusion (Figure 1). On day 5 of life, the newborn's respiratory status worsened; the respiratory distress required increased oxygen supplementations. The infant was intubated, and surfactant was administered. Viral and fungal cultures of the throat and trachea aspirates, and urine were obtained. Polymerase chain reaction (PCR) analyses for enteroviruses were negative. A cardiac echography performed and was normal. Another chest X-ray performed showed a diffuse alveolar pattern (Figure 2). Azithromycin, Vancomycin, and steroids were added to the treatment. On day 6 of life, due to a marked clinical deterioration of the infant's condition (tachypnea up to 80, a low mean blood pressure 38 mmHg) combined with worsening hypoxemia and hypercarbia and a high lactate level (21.6), high frequency ventilation and nitric oxide (NO) were initiated and dopamine treatment was given. Laboratory exams showed thrombocytopenia (42,000 cells/*µ*L), an increased C-reactive protein level (7 mg/L), and signs of liver failure (AST 3760 U/L and ALT 422 U/L) associated with a mild hypoalbuminemia (2.5 g/dL). A transfusion of plasma, packed red cells, and platelets was given, and antimicrobial therapy was upgraded, to Tazobactam. Despite full intensive care support including a prolonged cardiopulmonary resuscitation, the baby died of rapidly progressive multiple organ failure.

Post-mortem investigations were carried out. A PCR of nasopharyngeal and tracheal aspirates and a rectal swab demonstrated a positive HSV1 DNA PCR. Maternal history revealed a vaginal discharge before delivery. Her blood count before delivery showed a mild thrombocytopenia (105 cells/*µ*L) while her urine and stool cultures were negative.

## 3. Discussion

Neonatal HSV infection is increasingly becoming a major cause of morbidity and mortality in NICUs around the world [6, 7]. Addressing this condition is therefore a great challenge for the neonatologist.

The present case report is instructive for three main reasons. Firstly, neonatal HSV infection is an uncommon disease that yields nonspecific clinical manifestations, which may mislead the neonatologist, and therefore delay treatment initiation. The current case urges to use rapid diagnosis tools such as PCR for HSV, while investigating a severe pneumonia in a newborn. Inserting this investigation to our protocol could have saved the infant's life. Many NICUs around the world have adopted the recommendation [6–9] to start anti-viral treatment with Acyclovir in a neonate under 21 days with fever, pneumonia, and sepsis-like disease that is resistant to antibiotics, even when there are no cutaneous or neurological manifestations and in the absence of maternal herpetic lesions. Secondly, pneumonia is considered a component of disseminated neonatal HSV infection; however, some authors report cases of HSV pneumonia without manifestations of dissemination [4, 8, 10–12]. The case we describe is in our opinion, an example of primary HSV pneumonia in a newborn. We suggest that HSV pneumonia albeit uncommon should be deemed as a specific entity while investigating etiologies of pneumonia in the neonatal period. In addition, we propose a modification of the neonatal HSV infection classification by isolating HSV pneumonia as a specific pattern, similar to HSV CNS disease which is regarded as a distinct entity [5]. Thirdly, it is widely accepted that the overwhelming majority of neonatal HSV infections are caused by HSV2 [13]. However, many reports revealed that genital HSV1 is incriminated in serious cases of neonatal herpes infection [6, 14]. In our case HSV1 DNA was detected in nasopharyngeal and endotracheal aspirates, but the identification was too late to influence the outcome. Our patient presented with fever on day 3 of life (which can be considered as the onset of the disease) and his condition swiftly deteriorated until his demise 3 days later. This observation corroborates the findings of Capretti et al. [6], who described a patient that presented a severe disseminated HSV1 infection with necrotizing hepatitis and diffuse interstitial pneumonia. The rapid deterioration of our infant's condition demonstrates the severity of the HSV1 infection and the need for early aggressive treatment. Treatment of HSV infection with high-dose acyclovir has been shown to decrease mortality from 85% to 29%, if the disease is identified early and treatment is immediately initiated [5]. This highlights the necessity to consider vaginal HSV1 as a potential lethal agent. Consequently, this infection requires awareness from both neonatologists and pediatric infectious disease specialists and should be considered in newborns with an unidentified infectious disease, even in the absence of typical clinical presentation or typical blood tests such as thrombocytopenia and elevated liver enzymes.

## 4. Conclusion

In conclusion, our case report highlights neonatal HSV1 pneumonia as a primary and main manifestation of herpes infection. Increased awareness of neonatal HSV pneumonia is vital to achieve early treatment and successful outcome.
